# Effect of specific training course for competency in professional oral hygiene care in the intensive care unit: a quasi-experimental study for developing a standardized learning curve

**DOI:** 10.1186/s12871-022-01709-2

**Published:** 2022-06-01

**Authors:** Abbas Samim, Amir Vahedian-Azimi, Ali Fathi Jouzdani, Farshid Rahimi-Bashar

**Affiliations:** 1 Chemical Injuries Research Center, Systems Biology and Poisonings Institute, Baqiyatallah University of Medical Sciences, Tehran, Iran; 2 Trauma Research Center, Nursing Faculty, Baqiyatallah University of Medical Sciences, Tehran, Iran; 3 Student Research Committee, Hamadan University of Medical Sciences, Hamadan, Iran; 4 Anesthesia and Critical Care Department, Hamadan University of Medical Sciences, Ayatolah Motahari BLVD, Resalat Square, 6514845411 Hamadan, Iran

**Keywords:** Oral hygiene care, Nursing education, Learning curve, Intensive care unit

## Abstract

**Background:**

The development of evidence-based training standards can help improve the quality of educational programs for novice intensive care unit (ICU) nurses. This study was conducted to assess the application of a training course on competency development of nurses in relation to oral hygiene care in ICU patients and to develop a checklist for evaluating the competence performance. In addition, to achieve a certain level of oral hygiene competence, as well as to assess the relative importance of predicting factors and learning competency patterns in oral hygiene care, we used standard learning curve.

**Methods:**

This quasi-experimental study with time series design was conducted on newly registered ICU nurses of a teaching hospital affiliated with Tehran University of Medical Sciences, Iran, between 2016 and 2018. In the first phase of this study, we designed a checklist to assess nurses' professional competence in oral hygiene care in three stages: before, during, and after care. Then, in the second phase, the level of competence of nurses in repeated times of oral hygiene care was determined based on checklist items and recorded in the learning curve.

**Results:**

The greatest increase of oral hygiene care competency due to repetition was observed in the first and fourth times of repetition in comparison to the subsequent and previous steps. In the linear regression model, demographic variables predicted 12–19% of changes related to skill scores in repetitions.

**Conclusion:**

According to the learning curve, newly registered ICU nurses can reach an acceptable competency after 6 repetitions of oral hygiene care.

## Introduction

Effective provision of nursing intensive care affects the patient safety and patient satisfaction with care [1, 2]. The term “missed nursing care” refers to essential nursing care that is partially or completely delayed [3]. Certain detrimental results are associated with missed nursing care including higher occurrence of infections, delirium, pneumonia, prolonged hospitalization, malnutrition, as well as high levels of pain and discomfort [4–6]. Missed nursing care events cover a wide range of basic hospital nursing responsibilities, where one of the most frequently missed items identified in nursing is oral hygiene care in critically ill patients [7]. Oral hygiene in hospitalized patients, especially those in intensive care units (ICUs) is the essential element of care that helps maintain the health of critical ill patients [8]. ICU patients are completely dependent on nursing care due to their critical health conditions. Most of these patients are not able to perform the simplest activities such as oral hygiene due to unconsciousness or structural limitations of the ICU [9, 10]. Oral health problems in critically ill patients are often overshadowed by other serious needs in this population.

The safety of ICU patients may be threatened by a variety of pathogens that can lead to secondary complications [11]. In addition, mouth breathing and altered salivary flow due to various medications can lead to impaired oral hygiene and systemic secondary infections, such as ventilator-associated pneumonia (VAP) [12–15]. VAP as a nosocomial pneumonia is responsible for 25% of all nosocomial infections in ICUs, and is responsible for 9% to 13% of deaths in critically ill patients [16, 17]. However, evidence shows that maintaining oral hygiene in critically ill patients may reduce the risk of VAP by as much as 60% [18–20]. Although ICU nurses are aware of the importance of oral hygiene care and know that frequent care has a positive effect on health of patients, due to the need for repetition, methods employed and requisites used for care, its implementation has a low-priority [21, 22]. It seems that evidence-based training and practice are needed to improve techniques for professional oral hygiene care, patient outcomes, and cost-effectiveness [23, 24]. Evidence-based training and practice includes all aspects of nursing knowledge, attitudes, skills, and self-efficacy, which can help improve the quality of educational programs for novice ICU nurses [25]. To gain competence, the care process must be continuing until it is performed to standard criteria. For this purpose, the learning curve method can be used to examine the time required to acquire clinical competence, which indicates the improvement of individual skills in repeated experiences [26, 27].

Regarding the conditions of the intensive care unit for critically ill patients, the most modern equipment requires experienced personnel. Thus, policymakers expect nurses to be fully and independently prepared to take responsibility after repeating the care technique several times and gaining competence in doing. It is very important for policymakers to know how much training and practice is required for nurses to achieve professional care competence. Accordingly, we conducted this study to assess the application of a training course on competency development of nurses in relation to oral hygiene care in ICU patients and to develop a checklist for evaluating the competence performance. In this study, to achieve a certain level of oral hygiene competence, as well as to assess the relative importance of predicting factors and learning competency patterns in oral hygiene care, we used standard learning curve.

## Methods

### Study design

**Fig. 1:**
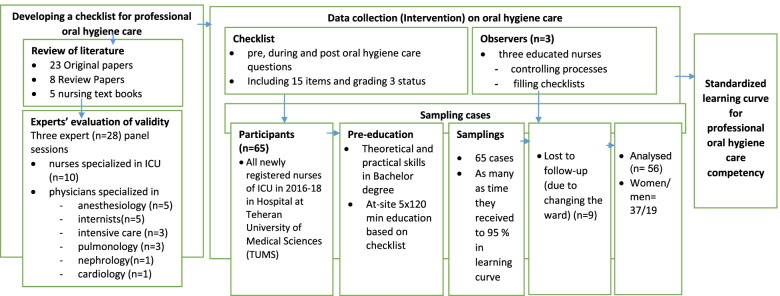
Study design for developing and testing a standardized learning curve for professional oral hygiene care competency for nurses

This quasi-experimental study with time series design is one of a series of research sets performed done to establish standard learning curves for various common care techniques in the ICU among newly registered ICU nurses in a teaching hospital affiliated of Tehran University of Medical Sciences, Iran, between 2016 and 2018. The first study "Effect of the Specific Training Course for Competency in Doing Arterial Blood Gas Sampling in the Intensive Care Unit: Developing a Standardized Learning Curve according to the Procedure’s Time and Socioprofessional Predictors" of this series of research has recently been published [28]. The current study was conducted to assess the application of a training course on competency development of nurses in relation to oral hygiene care in ICU patients and to create a checklist for evaluating the competence performance as well as for establishing standard learning curve for oral hygiene care. The study protocol was approved by the Ethics Committee of Hamadan University of Medical Sciences, Hamadan, Iran (IR.UMSHA.REC.1400.603), in accordance with the Declaration of Helsinki of the World Medical Association [29]. Further, the study was introduced with research ethics committee of Tehran University of Medical Sciences (TUMS), and the research package was explained clearly to hospital authorities. After expressing ethical considerations including principles of secrecy, confidentiality of personal information and privacy of the subjects, the objectives of the study were explained to all participants (nurses), and written informed consent was obtained from the nurses who agreed to be included in the study. This study was registered at U.S. National Library of Medicine "Clinicaltrials.gov" under code: NCT02830971, https://clinicaltrials.gov/ct2/show/NCT02830971, first dated 13–07-2016 with last updated on 15–04-2021. The study was conducted and reported in accordance with the recommendations of the Consolidated Standards of Reporting Trials (CONSORT) statement [30]. The study design for developing and testing a standardized learning curve for professional oral hygiene care competency for nurses is presented in Fig. 1.

### Participants

All newly registered nurses in ICU from 1 April 2016 until next 24 months from 10 mixed beds ICUs affiliated to a teaching hospital located in Tehran, Iran, were selected through convenience sampling and according to the inclusion criteria. The inclusion criteria in this study were (a) having a bachelor's degree in nursing, (b) being a newly registered nurse in the ICU, (c) no practical experience in performing oral hygiene techniques in the ICU, (d) not having a preventive physical disability to perform skills, and (e) willingness to participate in the study. Nurses were excluded from the study if they were transferred from the ICU to other wards during the study period.

### Sample size

The sample size was determined based on power analysis using G*Power software (latest ver. 3.1.9.7; Heinrich-Heine-Universität Düsseldorf, Düsseldorf, Germany), according to type I error of 5% to achieve a 95% confidence level and 90% power (www.psycho.uni-duesseldorf.de/abteilungen/aap/gpower3). Initially, 65 nurses who were recruited from the newly registered nurses participated in the study based on inclusion criteria. Nevertheless, during the study, 9 nurses were excluded from the study because of change to a ward duty, where ultimately, 56 nurses remained in the study and completed the skill course.

### Development of competency checklist

The checklist was developed and validated in three stages: (a) developing a preliminary version of the checklist based on literature findings and international guidelines, (b) holding three rounds of expert panels including ten highly experienced ICU nurses and 18 expert physicians (intensivists, anesthesiologists, pulmonologists, internists, nephrologist, cardiologist), for consensus on all items of the checklist, (c) validating the checklist by an expert panel. To prevent bias, the agreement among the members of panel at each session was estimated based on the Kendall's coefficient of concordance where the high value of Kendall's [K (range): 0.943–0.976, *P*-value < 0.0001] revealed a strong association between them. During the three validation sessions, the content validity ratio (CVR) was 0.56 with 28 panelists and the content validity index (CVI) was 0.89. The reliability of the checklist was assessed using the inter-rater reliability method with the Kappa agreement test by the principal investigator with all members of the expert panel. All details are available at https://clinicaltrials.gov/ct2/show/NCT02830971.

### Training and data collection

Although the newly graduated and registered ICU nurses who participated in the study had previously been taught theoretical and practical oral hygiene skills during their studentship period, performing the oral hygiene care procedure was the first time for them in ICU. Training according to the components of the final checklist was presented theoretically to all participants by a full-time ICU instructor in five 120-min sessions. The contents of the theoretical and practical training sessions are presented in Table 1. After completing the theoretical training, three trained observers from ICU staff monitored the oral hygiene care process by the ICU nurses who participated in the study and filled out checklists accordingly. To prevent the bias of observer presence and reduce the effect of observers on participants, the scores of the first two weeks were not considered for each participant until that the presence of observers would be normal for nurses. The number of times required to achieve competency in performing the oral hygiene care of patients was assessed based on the learning curve method. The end point of reaching a score of 95 was considered in the learning curve. The reason for choosing less than 5% diversity is to be equal to flattening the learning curve (the plateau section) [31, 32]. In addition, the following socio-demographic characteristics data were also recorded for each participant: age, gender, grade point average (GPA), pre-education shift work, cooperation, major satisfaction, and supervisor.

**Table 1:** The contents of the theoretical and practical training sessions

Sessions	Contents
1	(a) physiology and anatomy of the mouth; (b) Pathophysiology of oral diseases
2	(a) importance of oral hygiene care in the prevention of various diseases in ICU patients such as ventilator-associated pneumonia (VAP); (b) the role of the occurrence of various oral diseases due to poor oral hygiene on the outcomes of ICU patients
3	Introduction and explanation of the components of the final checklist in the steps before, during and after professional oral hygiene care (part I)
4	Introduction and explanation of the components of the final checklist in the steps before, during and after professional oral hygiene care (part II)
5	Perform the practical three-step professional oral hygiene care checklist on the model

### Statistical analysis

The statistical methods used in this study were similar to our previous study [28], which is part of a series of similar studies to establish standard learning curves for various common care techniques in the ICU among newly registered ICU nurses. Poisson regression model was used to examine the factors affecting the frequency of practice to achieve a certain level of oral health competence. Nine Poisson models were fitted to the data according to the practice times required to achieve skill scores (including 55, 60, 65, 70, 75, 80, 85, 90, and 95) with predictable GPA, gender, age, pre-education score, shift, cooperation, and supervisor. For each model (each cut-off point), the predicted values ​​of the model (aligned) were evaluated in contrast to the main values ​​and according to the following calculation: Accuracy percentage = ((Actual times = Rounded Predicted times) / sample size) × 100. The precision of the model in estimating the exercise times required until achieved a certain level of competence. Finally, the best Poisson model with the best precision for prediction and identification as well as the best results was reported [33]. The average practice time, boxplot, and learning level required to reach each cutoff point were for the high and low initial proficiency levels through the learning curves of the seven exercises. The nurse’s skills were categorized according to the average of the primary skills (those who were less than the average was labeled as low, and others were supposed to be high or on normal level). In addition, the relative importance of demographic variables in predicting exercise outcomes was calculated by R software linear regression using the relaimpo package. The relative importance is indeed the resolution of R2 in the linear regression model, showing the contribution of each variable to the total predicted changes where the sum of each of these effects is equal to R2 in the model. The larger the value sowed, the greater the importance and impact of the variable on the linear model. All analyses were performed using the R software and the relaimpo package.

## Results

### Participant’s characteristics

The study sample consisted of 56 newly registered ICU nurses with a mean ± standard division (SD) age of 24.6 ± 1.5 years old. The mean age of men and women was not significantly different (24.2 ± 1.9 vs. 24.8 ± 1.3, *P* = 0.137). Most nurses were female (n = 37, 66.1%), and the majority were right-handed (n = 50, 89.3%). The mean GPA of participants was 16.9 ± 1.4 from 20. No statistically significant difference was found between the mean scores of GPAs of nurses according to the gender.

### Final competency development checklist

The final checklist included 15 items in three areas, including pre-oral hygiene care (6 questions), during oral hygiene care (5 questions), and post-oral hygiene care (4 questions). The final checklist with three domains for the professional oral hygiene care is presented in Table 2. The evaluation of each question was based on 3 states; ability to perform alone (2 score), ability to perform with assistance (1 score), and inability to perform (0 score). Each question had a specific importance coefficient between 1 and 5. The philosophy of the different coefficients of the questions in three areas was related to the level of importance of the question. Thus, in addition to domain scores, each domain had a specific importance coefficient. The total number of points in the checklist resulted from multiplying the question number by the weighting coefficient of the question in percentage. Hence, the highest score on the checklist was 100%and the lowest was 0%.

**Table 2:** Final checklist with three domains for the professional oral hygiene care

Domains of checklist		importance coefficient
**Before** **Professional Oral hygiene Care**	Prepare the required equipment's for each individual patient at the time of professional oral hygiene care	1
	Teaching the patient how to do professional oral hygiene care regardless of her/his level of consciousness	4
	Wear disposable gloves	1
	Assess the adequacy of the endotracheal tube cuff pressure	4
	Giving position 45–60 degrees to the patient's head	4
	Examination of the patient's oral cavity	5
**During** **Professional Oral hygiene Care**	Wear latex gloves	1
	Moisturizing the patient's oral cavity with 2 cc 0.9% normal saline through syringe	5
	180-degree rotation of impregnated spatula with 0.9% normal saline in all four parts of the mouth^a^	5
	180-degree rotation of impregnated spatula with 0.2% chlorhexidine in all four parts of the mouth^a^	5
	Rinse the mouth with 0.2% chlorhexidine solution during suctioning of the oral cavity	5
**After** **Professional Oral hygiene Care**	Collect equipment and remove latex gloves	1
	Correct the patient's head and arranging the patient's position	3
	Do not eat, suck, or take any action that clears the 0.2% chlorhexidine solution from the surface of the mouth for at least 20 min	3
	Changing the endotracheal tube band if it gets dirty (intubation patients)	3

^a^Divide the mouth into four parts; Right Upper Quadrant, Left Upper Quadrant, Right Lower Quadrant and Left Lower Quadrant

### Learning curve findings

**Fig. 2:**
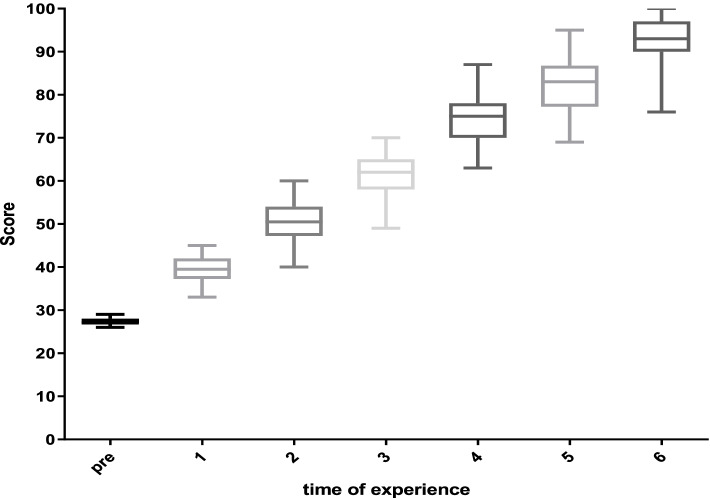
Box plot for scores for professional oral hygiene care

Figure 2 indicates the oral hygiene care skill scores based on the number of repetitions of the skill in the box plot design. The results show that more repetition leads to an increase in skill score, where 6 repetitions have led to a complete skill score. In each repetition of practices, it ended at the higher level of scores, including 30 as the first practice score, and it ended higher than 95 at the last practice repetition (6th). The maximum gained score of competencies in the 1st to 6th practices was 45, 60, 69, 85, 95, and 100, respectively. With increasing the practice time, the variation of skill scores increased where the range of scores in each practice time was totally different. Most changes in skill scores among nurses were observed in the 2th (from 45 to 60 score) and 4th (from 69 to 85 score) practices.

**Fig. 3:**
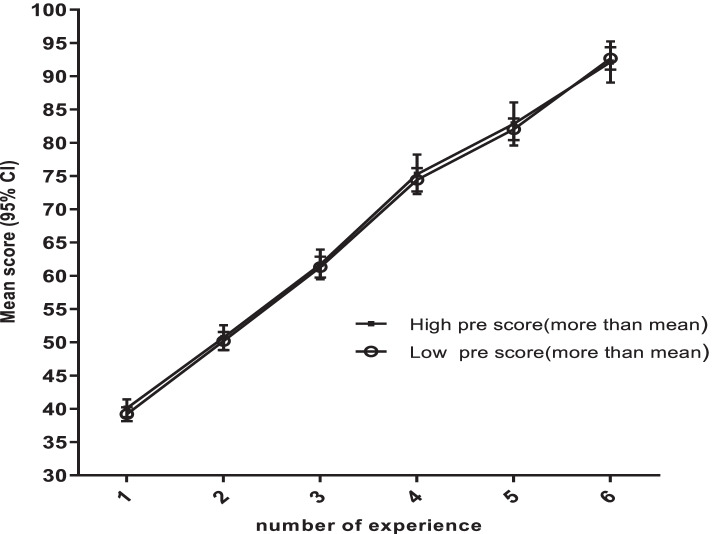
Mean score for professional oral hygiene care according to learning curve

Figure 3 displays the scores of oral hygiene care skill in the two groups according to the initial skill of participants (high or low score) from first to 6th time of repetitions. The initial skill of nurses was categorized according to the average of the primary skills (those who were less than the average were labeled as low, and others were supposed to be high level). The skill growth rate increased almost equally in both groups. It seems that the increase in oral hygiene care due to the number of repetitions in both groups was not related to the initial level of skill of nurses, where both groups reached a skill level with 6 repetitions. Only a slight difference was observed between the two groups in the level of nurses' skill in the 4th and 5th repetitions, in which nurses with higher initial skill score achieved slightly higher scores than nurses with lower initial skill.

**Fig. 4:**
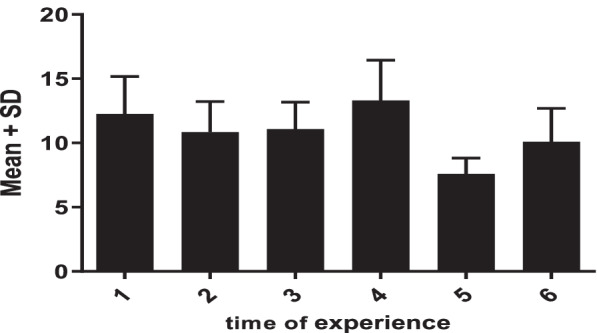
Mean changes in scores in professional oral hygiene care among times of experience

Figure 4 reveals the extent of increasing skill scores for each practice time, compared to the previous step. The effect of repetition of practice on increasing the competency of skill compared to the initial skill of nurses was very obvious; in the 2nd repetition, this effect decreased slightly, but in the 3rd and 4th repetitions, a significant effect on increasing skill was seen again. The mean difference between skill scores at 5th repetitions was decreased. However, the mean difference of skill score with 6 times of practice was increased compared with skill score with 5th repetitions. In general, the changes of competency values were not linear and mostly appeared in a sinusoidal shape (first decreasing, then increasing, and again decreasing thereafter).

**Fig. 5:**
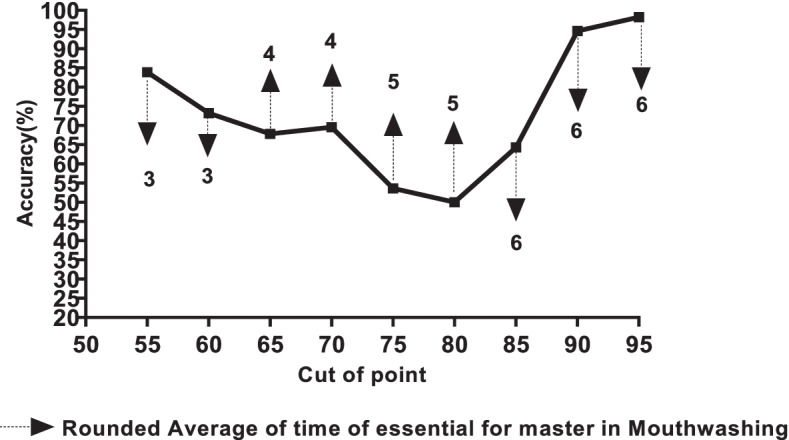
Poisson model accuracy in predicting the number of times essential for professional oral hygiene care according to the cutoff point of the learning curve

Figure 5 illustrates the average necessary times to reach the different levels of oral hygiene proficiency, which were reported at 6 measurement times for each nurse by the dashed line. According to the results, in order to reach the skill level from 55 to 60, 60 to 70, 70 to 80, and 80 to 95, nurses would need an average practice time of 3, 4, 5, and 6, respectively. In other words, with 3 practice times, nurses would reach the competency score of 60, with 4 practice times, they would reach the score of 70, and with 5 times of practice they would reach 80 score and by 6 times of practice they would reach the maximum skill score of 95.

**Fig. 6:**
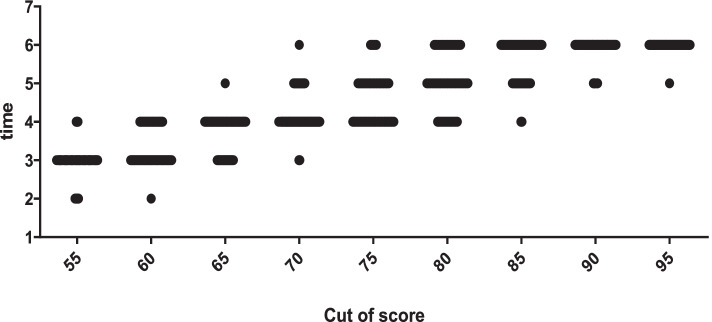
Distribution of time essential for professional oral hygiene care according to the cut-off point of the learning curve

Figure 6 depicts the scatter of necessary times to reach the different levels of competency by the segregation of nurses. According to this figure, to reach the scores of 55 to 60, 4 practice times were needed for the majority of nurses, and to reach a score of 65 to 70, 5 times of practice were required for the majority of the nurses. However, a small percentage of nurses reached 70 score even with three times of practice, while some of them (minority) even needed 5 or 6 practicing sessions to reach 80 points. To obtain 95 score, the majority of nurses needed 6 times of practice. However, a few numbers of nurses reached 95 score with 5 times of practice.

### Relative importance of predictor variables

According to Table 3 reporting the relative importance of predictor variables, demographic variables of nursed predict 12.02% of changes in related score in the first practice in multiple regressions according to R^2^. The extent of prediction of these variables was highest in the fourth practicing session (19.61%). On the other hand, at 5 and 6 times of practicing, it decreased to 17.82%. The major-satisfaction variable did not show a significant impact on the related score until at 2 and 3 times of practice. On the other hand, the impact of this variable increased by raising the number of practices. All variables had the predictability potential by the Poisson model and the results showed that the designed Poisson models with the mentioned variables with an almost good accuracy can predict necessary practice times to reach skill levels.

**Table 3:** Estimates of relative importance (decomposed R^2^) of predictors for the linear regression model under the entering method in R software

Covariate	Competency score time in professional oral hygiene care					
	**First**	**Second**	**Third**	**Fourth**	**Fifth**	**Sixth**
**Age**	0.016	0.020	0.005	0.030	0.020	0.018
**Gender**	0.002	0.008	0.002	0.007	0.009	0.027
**Marriage**	0.001	0.002	0.006	0.022	0.023	0.013
**BMI**	0.010	0.017	0.013	0.029	0.023	0.017
**Major Hand**	0.010	0.002	0.011	0.055	0.040	0.008
**GPA**	0.001	0.003	0.003	0.004	0.014	0.016
**Major Satisfaction**	0.006	0.072^a^	0.069	0.013	0.018	0.009
**Colleague's Cooperation**	0.008	0.008	0.022	0.021	0.020	0.026
**Present Supervisor**	0.000	0.012	0.002	0.002	0.000	0.027
**Shift**	0.010	0.009	0.001	0.007	0.008	0.012
**Pre-Education**	0.056	0.015	0.009	0.006	0.003	0.006
**R**^**2**^	12.02	16.63	14.34	19.61	17.82	17.82

^a^ 0.05

### Developing a standardized learning curve

**Fig. 7:**
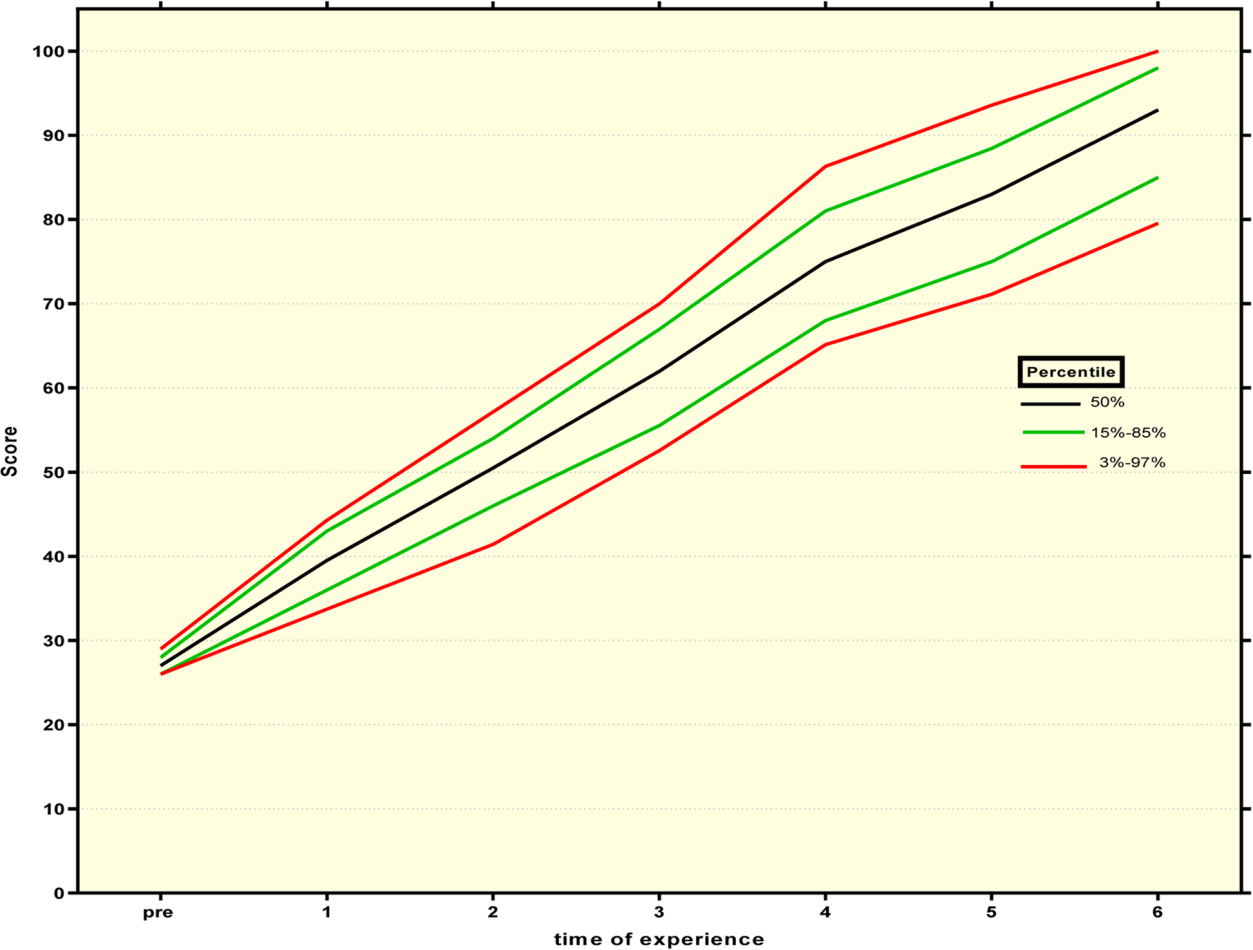
Percentile curve for scores for professional oral hygiene care. This graph presents the rate of increasing skill scores of nurses according to the number of practices. The black line indicated the median of scores, the red line indicated the percentile between 3 and 97%, and the green line indicated the percentile from 15 to 85%. The percentile from 3–15 is assumed the weak level, 15–50% is assumed the middle level, 50–85% is assumed a good level, and 85–90% is assumed a well level

Figure 7 indicates a percentile curve for assessing the ability of professional oral hygiene care. This graph shows the rate of increase in the nurse skill score with the number of practices. The black line represents the median score, the red line the 3% to 97% percentile, and the green line the 15% to 85% percentile. The range 3–15% percentile is considered a weak level, 15–50% a moderate level, 50–85% a good level, and 85–90% a good level. All variables had the predictability of a Poisson model, and the results almost accurately predicted the practice time required for a Poisson model designed using the above variables to reach skill levels 75–95. Achieving 75 score can be useless, and the accuracy of the Poisson model will be less than about 50%.

## Discussion

This study was one of the first studies in the field of nursing education that used the learning curve to evaluate the newly registered ICU nurses' procedural competence in oral hygiene care. In the first phase of this study, we designed a checklist to assess nurses' professional competence in oral hygiene care in three stages: before, during, and after care. Then, in the second phase, the level of competence of nurses in repeated times of oral hygiene care was determined based on checklist items and recorded in the learning curve. Our results indicated that the learning curve of newly registered ICU nurses in oral hygiene care reached a plateau level after 6 repetitions. The greatest increase of oral hygiene care competency due to repetition was observed in the first- and fourth-time’s repetition in comparison to the subsequent and previous steps. Significant increase in nurses' skill in the first repetition after the training compared to their initial skill suggests the importance of nurses' training and its effect on increasing their ability, which can enhance the confidence and satisfaction of new ICU nurses and encourage them to continue as well as repeat skill until reaching competency [34, 35].

ICU patients need specialized care with in-depth knowledge and skills that can ensure patient safety. Oral hygiene care is essential to prevent VAP in this population. Failure to perform regular and effective oral hygiene leads to increased levels and diversity of oral microflora that make up dental plaque [36]. Plaque on tooth surfaces, coated tongues, and periodontal disease exacerbate the patient's clinical condition as they provide a favorable environment for the growth of Gram-negative bacteria [37, 38]. Intensive care nurses are responsible for performing oral hygiene in critically ill patients in ICUs; so, the knowledge, attitude, and practice of nurses have a great impact on patients' health [39]. In confirmation of the results of this study, Park et al. [40] also showed that with laparoscopic resection surgery of colorectal cancer, participants' competence increased while the duration of surgery as well as its side effects decreased, especially in the early stages.

Our study also compared nurses' learning curve with respect to their initial skill level. The results indicated that the skill growth rate increased almost equally in both groups and finally the competency score reached 95 at 6th time of practice. Here, the nurses with higher initial skill in oral hygiene care did not have a significant advantage in learning and improving their skill level compared to less skilled nurses. Contrary to the results, in a previous study [28], we found that nurses with a lower initial skill in arterial blood gas (ABG) sampling required more time for repetition to achieve a maximum skill level score than nurses with higher initial skill. In a study by Loukas et al. [41], the learning curve of novices in intravenous cannulation reached a plateau level after 8 times, while in intermediates, it reached a plateau level after 6 times. This flexibility is an advantage of the learning curve method, which can be used to predict the expected frequency for obtaining competency at any level of initial skills of participants. Unfortunately, to date, no study has been conducted on the competence of oral health care in the ICU by learning curves method which can be compared with the results of this study.

This study indicated that the socio-demographic characteristic of nurses including age, sex, marital status, major hand, GPA, major satisfaction, colleague's cooperation, present supervisor, shift, and pre-education can predict 12–19% of changes in oral hygiene scores. Evidence suggests that the proficiency type and demographic characteristics could be effective on the extent of acquiring competency [42]. In addition, previous studies have shown that demographic factors such as age, sex, and ethnicity can predict success in medical education, but they did not mention the extent of the prediction [43, 44]. By considering these factors in the scoring levels of learning curve, the curve value could be improved for its utilization in similar learning environments.

Another important finding of this study was the creation of a standard learning curve for oral health care. The learning curve shows the progress of learning scores in oral health care (vertical axis) in repeated experiments (horizontal axis) through percentile lines. This curve is constantly rising and simply reveals the average training time required for each level of competency. Note that if oral hygiene car repetition were 6 times, 97% (up to the 3% percentile line) of people would reach 90–100 of skill scores, 70% (between 15 and 85% percentile lines) 95–100 of skill scores, 50% of people scores higher than 97, and less than 3% of people would reach skill scores lower than 90.

The use of learning curve model in nursing education may have some advantages such as standardization of education and its cost-effectiveness through repetition of procedures, reduction of iatrogenic injuries of patients due to excessive repetition of procedures and improving the quality of nurses' education with any level of initial skills. This scientific method is reusable and can be practiced for different medical skills in different educational environments and can help develop the level of training practice in the field of nursing. It is suggested that the learning curve be considered in future studies in standardizing learning of other skills. Lack of control group and random allocation of nurses to receive training for comparing the effectiveness of training between the two groups have been the limitations of this study.

## Conclusions

In the first phase of this study, we designed a checklist to assess nurses' professional competence in oral hygiene care in three stages: before, during, and after care. Then in the second phase, the level of competence of nurses in repeated times of oral hygiene care was determined based on checklist items and recorded in the learning curve. According to the learning curve, newly registered ICU nurses can reach an acceptable competency after 6 repetitions of oral hygiene care. Utilization of the learning curve could be helpful in the standardization of clinical practices in nursing education and the frequency of skill practice optimization, thus improving the education quality in this field.

## Data Availability

The data of this study are not publicly available for privacy reasons, however are available from the corresponding author upon reasonable request.

## Abbreviations

ICU: Intensive Care Unit
VAP: Ventilator-associated pneumonia
MV: Mechanical ventilation
CVR: Content validity ratio
CVI: Content validity index
GPA: Graduate point average

## References

[1] Hessels AJ, Flynn L, Cimiotti JP, Cadmus E, Gershon RR (2015). The impact of the nursing practice environment on missed nursing care. Clin Nurs Stud.

[2] Karaca A, Durna Z (2019). Patient satisfaction with the quality of nursing care. Nurs Open.

[3] Kalisch BJ, Landstrom GL, Hinshaw AS (2009). Missed nursing care: a concept analysis. J Adv Nurs.

[4] Recio-Saucedo A, Dall'Ora C, Maruotti A, Ball J, Briggs J, Meredith P, Redfern OC, Kovacs C, Prytherch D, Smith GB (2018). What impact does nursing care left undone have on patient outcomes? Review of the literature. J Clin Nurs.

[5] Hessels AJ, Paliwal M, Weaver SH, Siddiqui D, Wurmser TA (2019). Impact of patient safety culture on missed nursing care and adverse patient events. J Nurs Care Qual.

[6] Kalisch BJ, Xie B, Dabney BW (2014). Patient-reported missed nursing care correlated with adverse events. Am J Med Qual.

[7] Kalisch BJ, Landstrom G, Williams RA (2009). Missed nursing care: errors of omission. Nurs Outlook.

[8] Miranda AF, de Paula RM, de Castro Piau CG, Costa PP, Bezerra AC (2016). Oral care practices for patients in intensive care units: a pilot survey. Indian J Crit Care Med.

[9] Yang R (2016). Dependency in critically Ill patients: a meta-synthesis. Glob Qual Nurs Res.

[10] Diaz TL, Zanone SJ, Charmo-Smith C, Kamoun H, Barrais AI (2017). Oral care in ventilated intensive care unit patients: observing nursing behavior through standardization of oral hygiene tool placement. Am J Infect Control.

[11] Argov Z, Latronico N (2014). Neuromuscular complications in intensive care patients. Handb Clin Neurol.

[12] Oliveira MS, Borges AH, Mattos FZ, Semenoff TA, Segundo AS, Tonetto MR, Bandeca MC, Porto AN (2014). Evaluation of different methods for removing oral biofilm in patients admitted to the intensive care unit. J Int Oral Health.

[13] de Melo Neto JP, Melo MS, dos Santos-Pereira SA, Martinez EF, Okajima LS, Saba-Chujfi E (2013). Periodontal infections and community-acquired pneumonia: a case-control study. Eur J Clin Microbiol Infect Dis.

[14] Mummolo S, Nota A, Caruso S, Quinzi V, Marchetti E, Marzo G (2018). Salivary markers and microbial flora in mouth breathing late adolescents. Biomed Res Int.

[15] Vilela MC, Ferreira GZ, Santos PS, Rezende NP (2015). Oral care and nosocomial pneumonia: a systematic review. Einstein (Sao Paulo, Brazil).

[16] Galal YS, Youssef MR, Ibrahiem SK (2016). Ventilator-associated pneumonia: incidence, risk factors and outcome in paediatric intensive care units at Cairo University Hospital. J Clin Diagnostic Res..

[17] Timsit JF, Esaied W, Neuville M, Bouadma L, Mourvllier B (2017). Update on ventilator-associated pneumonia. F1000Research.

[18] Hua F, Xie H, Worthington HV, Furness S, Zhang Q, Li C (2016). Oral hygiene care for critically ill patients to prevent ventilator-associated pneumonia. Cochrane Database Syst Rev..

[19] Atashi V, Yousefi H, Mahjobipoor H, Bekhradi R, Yazdannik A (2018). Effect of oral care program on prevention of ventilator-associated pneumonia in intensive care unit patients: a randomized controlled trial. Iran J Nurs Midwifery Res.

[20] Choi E-S, Noh H-J, Chung W-G, Mun S-J (2021). Development of a competency for professional oral hygiene care of endotracheally-intubated patients in the intensive care unit: development and validity evidence. BMC Health Serv Res.

[21] Binkley C, Furr LA, Carrico R, McCurren C (2004). Survey of oral care practices in US intensive care units. Am J Infect Control.

[22] Benson CM, Maibusch R, Zimmer SE (1980). Oral health of hospitalized patients. Part 1: an overview of oral hygiene nursing care. Dental Hygiene..

[23] Farokhzadian J, Jouparinejad S, Fatehi F, Falahati-Marvast F (2021). Improving nurses' readiness for evidence-based practice in critical care units: results of an information literacy training program. BMC Nurs.

[24] Farokhzadian J, Nayeri ND, Borhani F, Zare MR (2015). Nurse leaders' attitudes, self-efficacy and training needs for implementing evidence-based practice: is it time for a change toward safe care?. Brit J Med Medic Res.

[25] Kronenfeld M, Stephenson PL, Nail-Chiwetalu B, Tweed EM, Sauers EL, McLeod TC, Guo R, Trahan H, Alpi KM, Hill B (2007). Review for librarians of evidence-based practice in nursing and the allied health professions in the United States. J Med Library Assoc.

[26] Barrington MJ, Viero LP, Kluger R, Clarke AL, Ivanusic JJ, Wong DM (2016). Determining the learning curve for acquiring core sonographic skills for ultrasound-guided axillary brachial plexus block. Reg Anesth Pain Med.

[27] Nguyen BV, Prat G, Vincent JL, Nowak E, Bizien N, Tonnelier JM, Renault A, Ould-Ahmed M, Boles JM, L'Her E (2014). Determination of the learning curve for ultrasound-guided jugular central venous catheter placement. Intensive Care Med.

[28] Vahedian-Azimi A, Rahimi-Bashar F, Pourhoseingholi MA, Salesi M, Shamsizadeh M, Jamialahmadi T, Gohari-Moghadam K, Sahebkar A (2021). Effect of the specific training course for competency in doing arterial blood gas sampling in the intensive care unit: developing a standardized learning curve according to the procedure's time and socioprofessional predictors. Biomed Res Int.

[29] World Medical Association (2013). Declaration of Helsinki: ethical principles for medical research involving human subjects. JAMA.

[30] Jayaraman J (2020). Guidelines for reporting randomized controlled trials in paediatric dentistry based on the CONSORT statement. Int J Paediatr Dent.

[31] Waldman JD, Yourstone SA, Smith HL (2003). Learning curves in health care. Health Care Manage Rev.

[32] Pusic MV, Boutis K, Pecaric MR, Savenkov O, Beckstead JW, Jaber MY (2017). A primer on the statistical modelling of learning curves in health professions education. Adv Health Sci Educ Theory Pract.

[33] Groemping U (2006). Relative Importance for linear regression in R: the package relaimpo. J Stat Softw.

[34] Bhagat V, Hoang H, Crocombe LA, Goldberg LR (2020). Incorporating oral health care education in undergraduate nursing curricula - a systematic review. BMC Nurs.

[35] Dagnew ZA, Abraham IA, Beraki GG, Mittler S, Achila OO, Tesfamariam EH (2020). Do nurses have barriers to quality oral care practice at a generalized hospital care in Asmara, Eritrea? A cross-sectional study. BMC Oral Health.

[36] Khasanah IH, Sae-Sia W, Damkliang J (2019). The effectiveness of oral care guideline implementation on oral health status in critically Ill patients. SAGE Open Nurs.

[37] Sharma N, Bhatia S, Sodhi AS, Batra N (2018). Oral microbiome and health. AIMS Microbiol.

[38] Seneviratne CJ, Zhang CF, Samaranayake LP (2011). Dental plaque biofilm in oral health and disease. Chin J Dent Res.

[39] Sreenivasan VPD, Ganganna A, Rajashekaraiah PB (2018). Awareness among intensive care nurses regarding oral care in critically ill patients. J Indian Soc Periodontol.

[40] Park IJ, Choi GS, Lim KH, Kang BM, Jun SH (2009). Multidimensional analysis of the learning curve for laparoscopic colorectal surgery: lessons from 1,000 cases of laparoscopic colorectal surgery. Surg Endosc.

[41] Loukas C, Nikiteas N, Kanakis M, Moutsatsos A, Leandros E, Georgiou E (2010). A virtual reality simulation curriculum for intravenous cannulation training. Acad Emerg Med Off J Soc Acad Emerg Med.

[42] Speelman CP, Kirsner K (2001). Predicting transfer from training performance. Acta Physiol (Oxf).

[43] Ferguson E, James D, Madeley L (2002). Factors associated with success in medical school: systematic review of the literature. BMJ.

[44] Lumb AB, Vail A (2004). Comparison of academic, application form and social factors in predicting early performance on the medical course. Med Educ.

